# Study on the Effect of Calcium Alloy on Arsenic Removal from Scrap-Based Steel Production

**DOI:** 10.3390/ma16083113

**Published:** 2023-04-15

**Authors:** Hanjie Yao, Changling Zhuang, Changrong Li, Song Xiang, Xiang Li, Guangkai Yang, Zezhong Zhang

**Affiliations:** 1 College of Material and Metallurgy, Guizhou University, Guiyang 550025, China; 2 Key Laboratory of Metallurgical Engineering and Process Energy Saving of Guizhou Province, Guiyang 550025, China; 3 Guizhou Institute of Technology, College of Materials & Metallurgical Engineering, Guiyang 550025, China

**Keywords:** arsenic removal, calcium alloy, inclusion, arsenic removal agent

## Abstract

Scrap steel is a kind of resource that can be recycled indefinitely. However, the enrichment of arsenic in the recycling process will seriously affect the performance of the product, making the recycling process unsustainable. In this study, the removal of arsenic from molten steel using calcium alloys was investigated experimentally, and the underlying mechanism was explored based on thermodynamic principles. The results show that the addition of calcium alloy is an effective means of reducing the arsenic content in molten steel, with the highest removal percentage of 56.36% observed with calcium aluminum alloy. A thermodynamic analysis revealed that the critical calcium content required for arsenic removal reaction is 0.0037%. Moreover, ultra-low levels of oxygen and sulfur were found to be crucial in achieving a good arsenic removal effect. When the arsenic removal reaction occurs in molten steel, the oxygen and sulfur concentrations in equilibrium with calcium were $w_{\left[O\right]}=0.0012\%$ and $w_{\left[S\right]}=0.00548\%$, respectively. After successful arsenic removal, the arsenic removal product of the calcium alloy is Ca_3_As_2_, which usually does not appear alone. Instead, it is prone to combining with alumina, calcium oxide, and other inclusions to form composite inclusions, which is beneficial for the floating removal of inclusions and the purification of scrap steel in molten steel.

## 1. Introduction

In recent years, short-process electric arc furnace (EAF) steelmaking has been increasingly adopted, replacing the traditional long-process steelmaking method of a “blast furnace-converter” due to its low carbon emissions and environmentally friendly production. Short-process EAF steelmaking currently accounts for approximately 30% of global steel production [1,2,3]. An electric arc furnace (EAF) is a furnace that utilizes an electric arc to provide heat for the heating and melting of waste materials. The temperature in the arc zone can reach up to 3000 °C, with the melting temperature around 1500 °C [4]. Using electric arc furnaces for short-process steelmaking not only utilizes a large amount of slag, but they can also reduce two-thirds of the pollution emissions. In addition, in line with policies and targets to reduce carbon emissions, China aims to significantly increase the utilization of scrap in the steel industry in the coming decades. Compared to long-process steelmaking, the use of scrap eliminates highly polluting and high-emission production stages, such as coking, sintering, and ironmaking. Therefore, the promotion of electric arc furnaces is widely considered an essential measure to achieve emission reduction in the steel industry [5,6,7,8,9].

Despite the benefits of short-process electric arc furnace (EAF) steelmaking, there exist challenges that need to be addressed in large-scale scrap utilization. While scrap steel is theoretically a recyclable raw material, the reality is that scrap sources are complex and contain numerous impurities. Some impurities are difficult to remove during production and gradually accumulate with each scrap steel cycle, eventually leading to a high impurity content that renders the scrap steel unusable and obstructs the infinite cycle of scrap steel. Arsenic is a typical impurity in scrap steel, which is challenging to remove in the production process, and there is no production process for arsenic removal [10,11,12,13,14,15]. The arsenic content accumulates with the circulation. Once the arsenic content in the molten steel exceeds the standard, the properties of the steel will be significantly reduced, and it can even directly lead to unqualified products. The literature shows that arsenic accumulates on the grain boundaries of steel, leading to temper brittleness. Hot brittle cracks occur easily in arsenic-containing steel during hot processing. When the impurity arsenic exceeds a certain content, it will cause severe segregation of components, form granular compounds with low hardness, reduce weldability and impact resistance, and reduce steel strength and plasticity [16,17,18,19,20,21]. Considering the recycling of steel products and the imperfect classification and treatment technology of scrap steel, the residual element arsenic in steel will be continuously enriched and the impurity arsenic in steel will cause an increasing number of adverse effects.

In addition, the long-term stacking of scrap steel will aggravate environmental pollution. Hazardous materials such as arsenic can seep into the soil, contaminate groundwater, and release into the atmosphere, posing significant risks to human health and the environment [12,22,23,24,25,26,27,28].

In the steel production process, residual element arsenic is controlled by metallurgical workers during both the iron ore treatment stage and the steelmaking production stage. Their approach involves reducing the total amount of harmful impurities during steelmaking and taking measures to suppress the harm caused by such elements. Various methods are employed, including batch dilution, iron ore reduction roasting, the removal of hot metal or liquid steel, and the addition of inhibitors. The ingredient dilution method uses high-quality molten iron to dilute the molten iron with high impurities to reduce the impurity content in the molten steel. However, more is needed to fundamentally solve the problem. The iron ore reduction roasting method converts the metal oxide in the ore into a corresponding low-valent metal compound or metal under the conditions of a lower melting point of the furnace charge and a reducing atmosphere. This method removes impurities that are studied more at present. However, it has a high cost and energy consumption and is difficult to handle, so it cannot be applied to large-scale industrialization. The method of removing hot metal or liquid steel is to control the elements of steel by pretreatment with hot metal or steelmaking treatment, which can remove the impurities of steel and recover valuable elements. Adding inhibitors to molten steel is a feasible method. It is also easy to control by adding inhibitory elements to improve the occurrence of impurities in molten steel and inhibit their segregation [29,30,31,32,33,34].

Yasushi et al. [35] conducted experiments to remove arsenic from 18%Cr-8%Ni steel using Ca-CaF_2_ slag and reported the successful reduction of arsenic. However, the utilization of calcium metal as an arsenic removal agent is hindered by its scarcity and high cost, and complicated control measures required for dearsenication. Katayama [36] found that CaC_2_-CaF_2_ slag can reduce the arsenic content in Fe-Cr alloy melt. However, the use of CaC2 as an arsenic removal agent is risky due to its severe reaction. Zhu et al. [37,38] used a CaC_2_-CaF_2_ slag system as an arsenic removal agent to study the reduction of arsenic in molten iron in the laboratory. The results show that it is impossible to simultaneously remove sulfur, phosphorus, and arsenic in the CaO-CaF_2_ slag system. In molten steel, arsenic can only be reduced, not oxidized. Dong et al. [39] further found that CaO-CaF_2_ can also be used as a dearsenication agent to effectively reduce the content of sulfur and arsenic in molten steel. Combined with the thermodynamics of Zhu et al. [37,38], the reason why phosphorus cannot be removed during the reduction of arsenic is further explained. Liu et al. [17] used the Si-Ca alloy-CaF_2_ to remove arsenic from molten steel, and the effect was worse than that of removing arsenic from molten iron under the same conditions. Li et al. [32,40] used the Si-Ca-Ba alloy-CaF_2_ as a dearsenication agent to study the dearsenication of liquid steel, with an initial arsenic content of 0.015–0.097% and an initial sulfur content of 0.005%, in a tube furnace, but did not consider the effectiveness of high-quality steel. In addition, some scholars use rare earth to study arsenic removal. Wang et al. [41] used rare earth lanthanum to remove arsenic from molten steel and studied the inclusions. It was found that the arsenic inclusions were mainly cluster-shaped La-S-As, and their composite inclusions combined with LaS and LaAs. La-S-As is a solid solution produced by randomly replacing the S atom of LaS with As, and its chemical formula is proved to be La_4_S_3_As. Xin et al. [31] studied the interaction between cerium and arsenic. The results show that the interaction between cerium and arsenic can produce different arsenic-containing rare earth inclusions. Ce-As inclusions can be non-uniformly nucleated with the preferentially formed rare earth inclusions as the core during solidification and can also be nucleated separately in molten steel. Combined with the research of scholars on the removal of arsenic in molten steel, they used different arsenic removal methods (Ca-CaF_2_, Si-Ca alloy-CaF_2_, lanthanum, etc.) to achieve the same. Although these methods have a good arsenic removal effect, they have not yet been applied in industrial production. These problems have also plagued metallurgical scholars and there has been no unique arsenic removal process in factory production.

In order to control the harm of arsenic in H08-3 electrode steel, studying the evolution of arsenic and inclusions containing arsenic in molten steel will help to understand the mechanism of arsenic in steel. Based on existing research, the effects of three different calcium-based alloys on the arsenic removal of high-quality electrode steel H08-3 were studied. The thermodynamic mechanism of arsenic removal was revealed by thermodynamic theory. The changes in oxygen, sulfur, arsenic, and inclusions in H08-3 electrode steel after adding the arsenic removal agent of the calcium alloy were analyzed to obtain a theoretically favorable condition for arsenic removal. Through an in-depth investigation of the mechanism of arsenic removal, this study provides a theoretical foundation for the removal of arsenic during steelmaking. By doing so, it has the potential to overcome the challenges of scrap recycling, increase the use of scrap in the steelmaking process, decrease the emission of pollutants, and ultimately contribute to environmental protection.

## 2. Experiments

### 2.1. Experimental Materials

Arsenic-containing H08-3 electrode steel was prepared using a vacuum induction heating furnace (Santai Electric Furnace Factory, Jinzhou, China). The specific process is: first, put the pure iron rod into the crucible according to the ratio and perform vacuum heating smelting. Once the metal in the steel is molten, add arsenic trioxide to the feeding channel to add arsenic to the molten steel. The arsenic-containing H08-3 steel obtained by smelting was used as the experimental base metal containing arsenic. A carbon sulfur instrument determined the C and S elements in the experimental base metal (Yanrui Instrument Co., Ltd., Chongqing, China). Inductively coupled plasma atomic emission spectrometry (ICP) determined the Si, Mn, P, and As elements in the experimental base metal (Huapu General Technology Co., Ltd., Shenzhen, China).

The experimental base metal obtained by smelting was cut with 0.1 mm molybdenum wire and, finally, several steel samples were obtained with a size of 10 mm × 10 mm × 30 mm. Table 1 shows the standard chemical composition of typical H08-3 steel for unique weathering and cryogenic vessel welding materials, and the composition of the materials selected in this experiment. The chemical composition content of standard H08-3 steel and test base metal is shown in Table 1.

### 2.2. Add Arsenic Removal Agent for the Arsenic Removal Experiment

A total of three arsenic removal experiments were carried out in the steelmaking furnace using a tubular resistance furnace. The experiment was carried out with an arsenic-containing H08-3 steel sample; three different calcium alloy dearsenication agents: (Ca-Fe alloy (Ca30%, Fe70%), Ca-Al alloy (Ca30%, Al70%), Ca-Si alloy (Ca30%, Si70%)); and aluminum powder (99.99%) as the primary experimental raw materials.

First, we added 350 g of arsenic-containing steel sample to the alumina crucible (OD48 mm × ID38 mm × HT110 mm), followed by coating with graphite crucible (OD70 mm × ID58 mm × HT190 mm). When the temperature increased to 600 °C, high-purity argon (purity 99.99%) was introduced for protection, and the flow rate was 2.2 L.min^−1^.When the temperature increased to 1500 °C, the argon flow rate was adjusted to 4 L.min^−1^. When the temperature increased to 1600 °C, the temperature was kept constant for 15 min. The aluminum powder was then added to the molten steel and stirred with an alumina rod for 10 min to fully dissolve the aluminum powder and allow the reaction to occur, after which it was left standing for 5 min. During the standing process, the quartz tube (OD8 mm × ID5 mm) was used to sample and cool as the initial deoxidized steel sample, which was recorded as H1. At a constant temperature of 30 min, the dearsenication agent was added to the molten steel five times; each time, it was stirred for 3 min until the agent was ultimately added and stood for 5 min (so that the dearsenication product floated). In the experiment, it is necessary to continuously stir the molten steel with an alumina rod to accelerate the arsenic removal reaction. After the arsenic removal product floated, a sample was taken every 5 min with a quartz tube, numbered H2-H6. After taking the sample, according to the pre-set procedures, the furnace temperature dropped to 900 °C. Here, we turned off the argon and power.

### 2.3. Composition Determination and Characterization of Non-Metallic Inclusions

The content of As in steel was determined by inductively coupled plasma atomic emission spectrometry (ICP) (Huapu General Technology Co., Ltd., Shenzhen, China). The content of the S element was determined by Yanrui Instrument Co., Ltd., Chongqing, China. An oxygen and nitrogen analyzer (Yanrui Instrument Co., Ltd., Chongqing, China) was used to determine the content of the O element.

The coarse grinding, fine grinding, and polishing samples were washed and dried with ethanol. The morphology and size of the inclusions were observed by the Zeiss-Utra55 field emission scanning electron microscope (Zeiss, Oberkochen, Germany). The composition of the inclusions was determined by an energy dispersive spectrometer (EDS) (Zeiss, Oberkochen, Germany). After a thorough understanding of the morphology of various inclusions, the morphology, size, and type of inclusions were observed under an optical microscope. Then, an optical microscope was used to take 100 consecutive images of the metallographic sample at a magnification of 2000 times. The optical microscope images were processed with Image-Pro Plus (Image-Pro Plus 6.0, Media Cybernetics, Rockville, MD, USA) image processing software to obtain information such as inclusion size and number density distribution. The characteristic parameters of the area of the inclusions were exported to the Excel table and the area of the inclusions in each metallographic image summed up separately. The area density of the inclusions in each picture was obtained by dividing the sum of the inclusion area by the area of the area taken by the metallographic image. We inserted a matrix in the origin drawing software, imported the area density value of the inclusions in each metallographic image into the matrix, and produced a three-dimensional rendering [42].

## 3. Results and Discussion

### 3.1. Experimental Results of Different Arsenic Removal Agents

**Figure 1:**
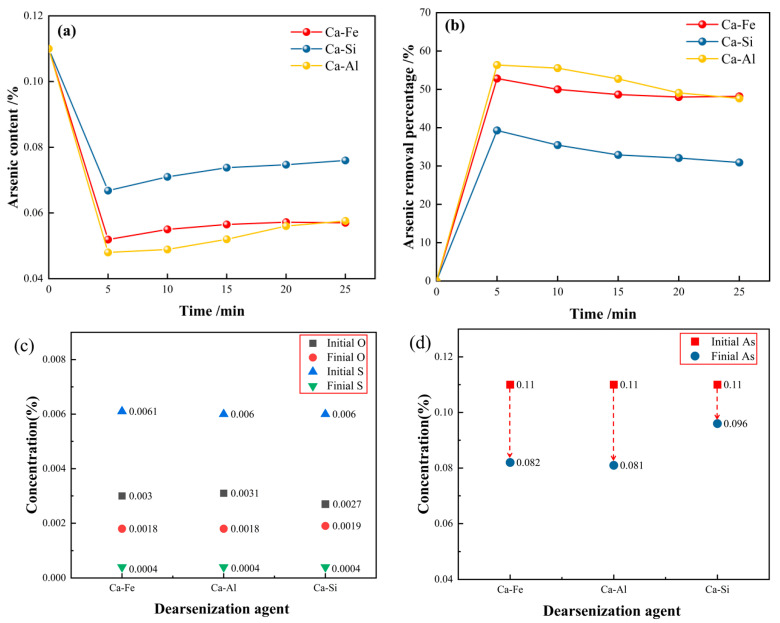

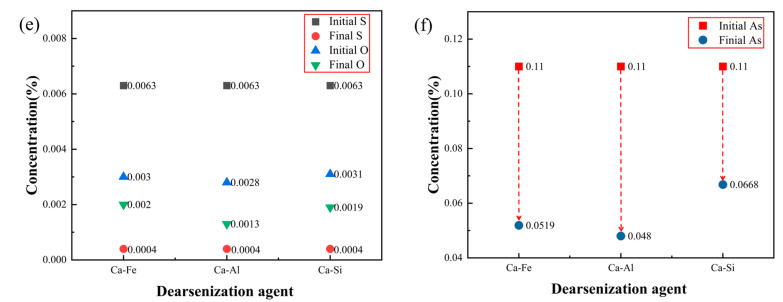
(**a**,**b**) Time varying curve of arsenic content and arsenic removal percentage; (**c**–**f**) change of chemical element content in steel.

According to the above experiments, the change in the arsenic content can be obtained using different arsenic removal agent experiments, as shown in Figure 1. It can be seen from Figure 1a that the arsenic content decreased significantly after adding three different calcium alloy dearsenizers. The arsenic content reached the lowest when the reaction was carried out for 5 min. The arsenic removal percentage formula is as follows [43]:

(1) $$\eta =\frac{{\left[\%As\right]}_{0}-{\left[\%As\right]}_{f}}{{\left[\%As\right]}_{0}}\times 100\%$$

where η is the percentage of arsenic removal; ${\left[\%As\right]}_{0}$ is the initial arsenic content; ${\left[\%As\right]}_{f}$ is the endpoint arsenic content. According to the ICP test, the arsenic content of the three experiments of calcium alloy dearsenifiers was measured, as shown in Table 2 below.

The following Table 3 shows the initial content and the endpoint content of oxygen, sulfur, and arsenic in steel using different experiments with calcium alloy dearsenication agents.

Figure 1b is the change curve of the arsenic removal percentage over time after adding three different calcium alloy dearsenication agents. The percentage of arsenic removal was highest when the reaction is carried out for 5 min. After 5 min, the arsenic content increased slightly, possibly due to the thermal decomposition of the arsenic removal product Ca_3_As_2_. Within 0–5 min after the reaction, the percentage of arsenic removal was fast, and the arsenic content decreased to a minimum, after which the arsenic content remained unchanged. The three calcium alloy arsenic removal agents used in this experiment achieved a good arsenic removal effect. When the reaction was carried out at 5 min, the arsenic removal percentage of the calcium aluminum alloy was 56.36%, the arsenic removal percentage of calcium iron alloy was 52.82%, and the arsenic removal percentage of the calcium silicon alloy was 39.27%. Therefore, the removal of arsenic in molten steel using a calcium aluminum alloy is the best, followed by a calcium iron alloy; the effect of the calcium silicon alloy is poor.

Figure 1c,d are experiments in which the dearsenication agent is directly added without deoxidizing the molten steel; Figure 1e,f are the experiments of the dearsenication of molten steel by adding the dearsenication agent after the addition of aluminum powder for the deoxidation treatment. Compared to Figure 1e,f, when the initial arsenic content is 0.11%, the arsenic content of the endpoint in Figure 1f is much lower than that in Figure 1d. To achieve a good arsenic removal effect, the molten steel must be deoxidized to control the oxygen content to a deficient range. It can be seen from Figure 1e that the oxygen content was controlled below 0.002% in this experiment, and a good arsenic removal effect was achieved. The oxygen content of the calcium aluminum alloy reached 0.0013%. The arsenic content at the endpoint arsenic was the lowest at only 0.048%, and the arsenic removal effect was the best. It can be concluded that the lower the oxygen content in the molten steel, the more favorable the removal of arsenic.

### 3.2. Dearsenication Products

**Figure 2:**
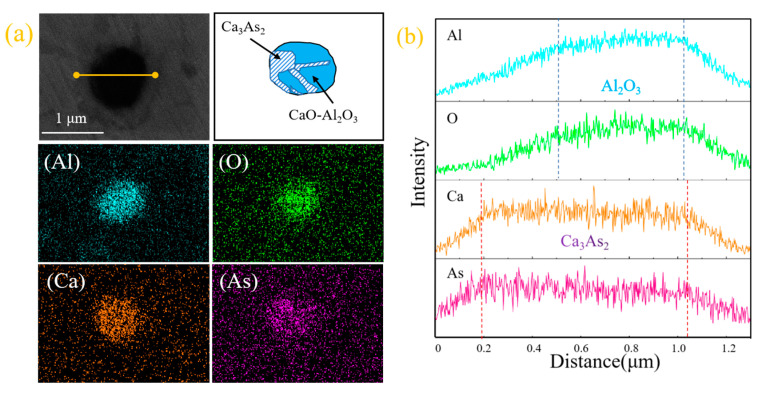

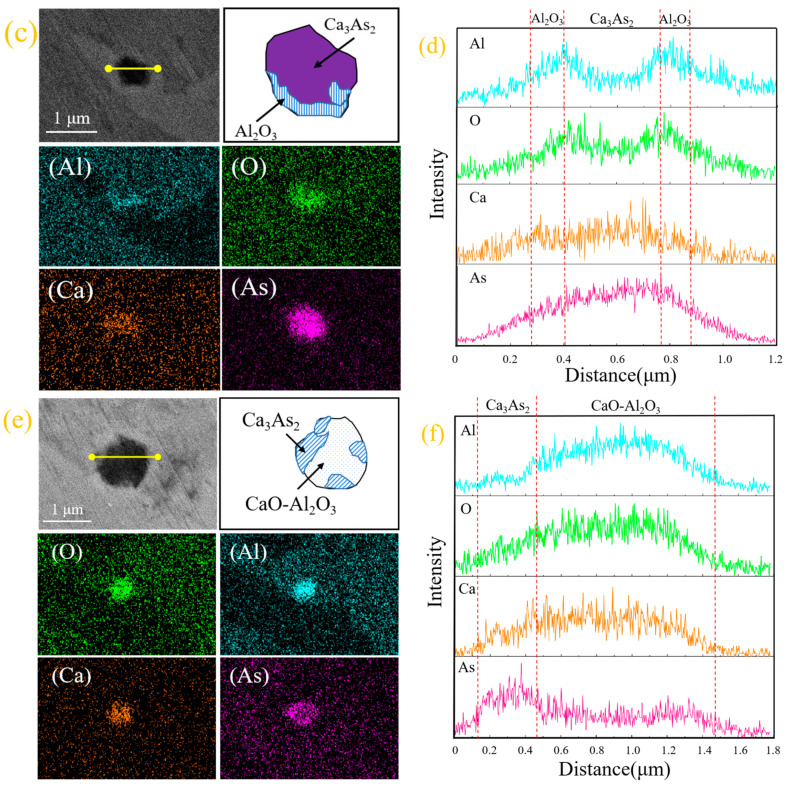
(**a**,**c**,**e**) Electron microscopy and morphology of inclusions; (**b**,**d**,**f**) line scan images of inclusions.

**Figure 3:**
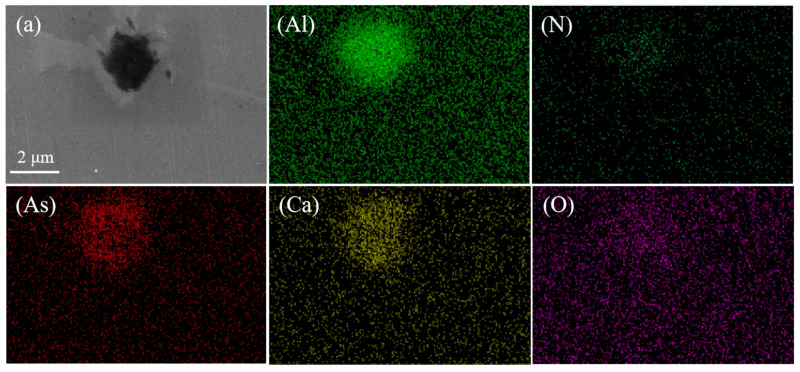

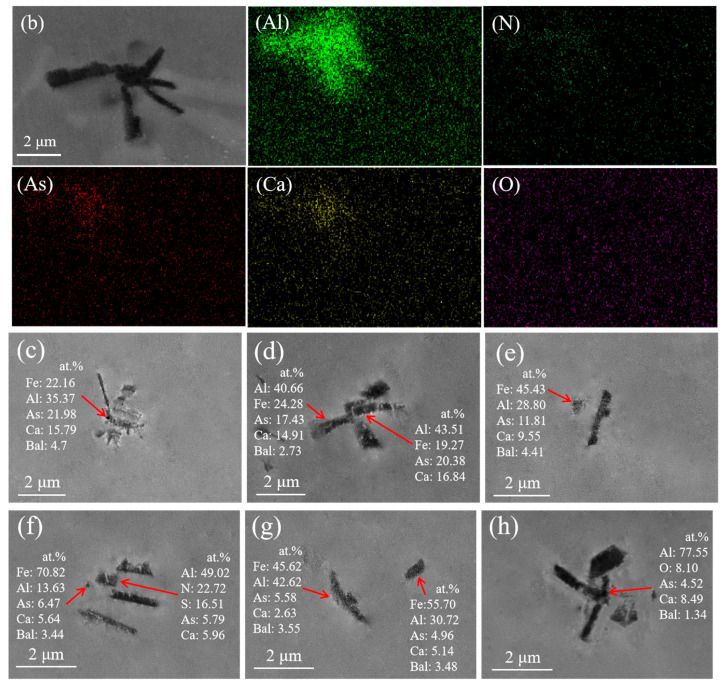
Arsenic inclusions and surface scan images. (**a**,**b**) Electron microscopy and morphology of inclusions. (**c**–**h**) Morphology and chemical composition of arsenic inclusions.

According to the field emission scanning electron microscope, we observed the arsenic-containing inclusions in the steel sample, as shown in Figure 2. It can be seen from the surface scanning and line scanning images of Figure 3a,b that this type of arsenic-containing inclusion is composed of dearsenication products Ca_3_As_2_ and calcium aluminate (xCaO-yAl_2_O_3_), and the dearsenication products and oxides are fused. Simultaneous precipitation of Ca_3_As_2_ and Al_2_O_3_ can form the inclusions of the compound.

The line scan image shows that this type of arsenic removal product is mainly concentrated in Ca_3_As_2_ and surrounded by inclusions of Al_2_O_3_. Furthermore, we exclude possible CaO and As_2_O_3_ inclusions. The comprehensive inclusion line scan and surface scan show that the inclusions appear to be layered structures: the outside is mainly Al_2_O_3_, and the inside is mainly Ca_3_As_2_; therefore, inclusions of Ca_3_As_2_-Al_2_O_3_ are formed. In light of this, this phenomenon may be due to Al_2_O_3_ and Ca_3_As_2_ adsorbed together in the process of the temperature drop.

From the scanning image of Figure 2e,f, it can be seen that the small part around this type of inclusion is Ca_3_As_2_ and the middle is calcium aluminate (xCaO-yAl_2_O_3_), thus forming a collision-type Ca_3_As_2_-xCaO·yAl_2_O_3_ inclusion. The formation of this inclusion may be due to the adsorption and collision of the dearsenication product Ca_3_As_2_ around the oxide to the calcium aluminate (xCaO-yAl_2_O_3_) during the temperature drop process, after the addition of the calcium alloy.

**Figure 4:**
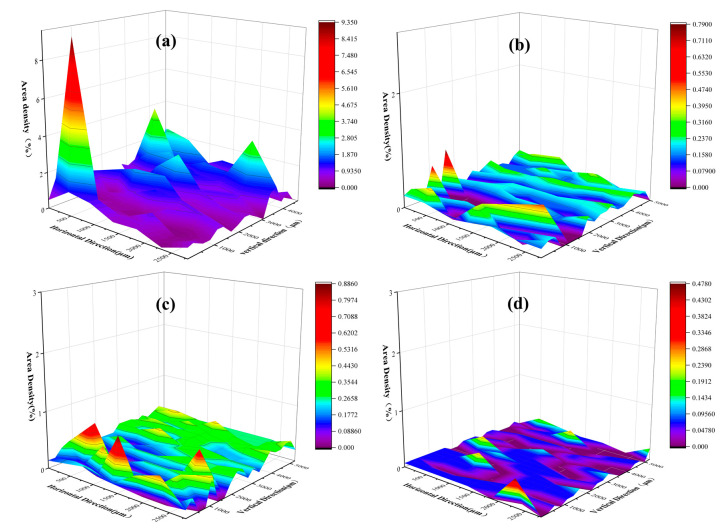
Change rule of inclusion area density distribution before and after dearsenication treatment of electrode steel. (**a**) is the area density distribution of inclusions in electrode steel before dearsenication; (**b**–**d**) is the area density distribution of inclusions after dearsenication treatment with ferro-calcium alloy, calcium–aluminum alloy and calcium–silicon alloy, respectively.

In addition, we still find a typical arsenic removal product diagram in the steel sample. Figure 3 shows the arsenic-containing inclusions and surface scanning images. It can be seen that the types of arsenic-containing inclusions are mainly calcium–arsenic compounds, calcium aluminates, and AlN. In this study, according to the two-dimensional coordinates of the inclusions on the surface of the sample and the area of each inclusion, the percentage of inclusions per unit area of the steel matrix on the surface of the sample at different positions was obtained, as shown in Figure 4.

Figure 4a is the area density distribution of inclusions in arsenic-containing steel before the arsenic removal treatment, and Figure 4b–d are the area density distribution of inclusions after the arsenic removal treatment. It can be seen from Figure 4a that the inclusions in the electrode steel are unevenly distributed, and the area density of inclusions is prominent, up to 9%. It can be seen from Figure 4b–d that the surface density of inclusions is small after the arsenic removal treatment, and the maximum is not more than 1%. Figure 4d is the dearsenication treatment with calcium silicon alloy; the surface density of inclusions is the smallest, and the distribution is the most uniform. Figure 4b shows the use of calcium aluminum alloy for the arsenic removal treatment, and the area density of the inclusions is relatively large. The possible reason is that the aluminum in the alloy generates inclusions that affect the steel sample. In summary, the use of three calcium alloys can not only play a role in arsenic removal, but also reduce the number of inclusions in molten steel, as well as modify them.

## 4. Analysis and Discussion

### Thermodynamic Analysis

In order to clearly understand the mechanism and process of arsenic removal in steel, it is necessary to perform a thermodynamic analysis of inclusions in the steel. Arsenic is an impurity in steel. Its oxidation potential is lower than that of Fe and its affinity with O is weaker than that of Fe, which makes it difficult to oxidize and remove during steelmaking. Arsenic will continue to enrich steel with recycling when its content reaches a certain level and will seriously impact steel performance [41,44,45,46,47,48,49].

**Figure 5:**
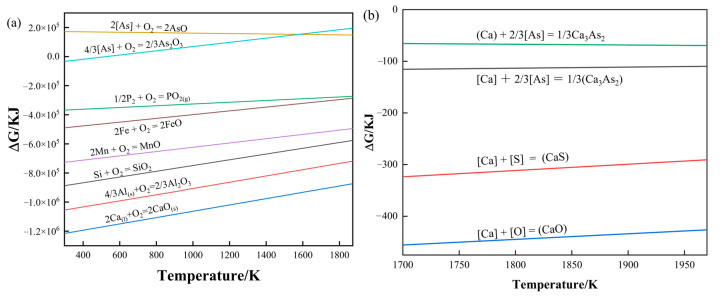
(**a**) Oxygen potential diagram of common elements in molten steel; (**b**) reaction of Ca with O, S, and As elements in molten steel.

Figure 5a is the oxygen potential diagram of common elements in molten steel. It can be seen from Figure 5a that the potential oxygen lines of the As and P elements are above the Fe element, which indicates that the binding ability of the As and P elements to oxygen in molten steel is weaker than that of the Fe element, so it is a challenge to oxidize and remove. The higher the oxygen potential line position, the more difficult it is to oxidize and remove it, and the As element cannot be oxidized and removed in the molten steel. Considering that the content of As element in molten steel is usually low, there is no arsenic removal process in the steelmaking process, which also causes the As element to remain in the steel [50,51]. From reactions (4)–(7), the Gibbs free energy and temperature curve can be drawn, as shown in Figure 5b. It can be seen from Figure 5b that when the molten steel is between 1773 K and 1873 K, the Ca dissolved in the molten steel can react with the elements O, S, and As in the molten steel. However, Ca reacts easier with O and S, and Ca and arsenic react with them more with greater difficulty. Therefore, to remove As in molten steel, the content of O and S in the steel should be controlled to a minimal level to facilitate the dearsenication reaction. This also explains why the dearsenication of calcium-based alloys after predeoxidation achieved better results than direct dearsenication.

The interaction coefficients of elements in liquid steel at 1873 K (1600 °C) are shown in Table 4 [32,52].

According to the Wagner model, the activity coefficient of elements in steel is calculated using the mass fraction of elements and interaction coefficient between elements in molten steel (see Table 4), and the activity of elements in molten steel is calculated using formulas (2) and (3) [53,54]:

(2) $$lgf_{i}=\sum_{}e_{i}{}^{j}\left[j\right]$$

(3) $$a_{\left[i\right]}=f_{i}\cdot \left[i\right]$$

$f_{i}$ is the activity coefficient of elements in steel; $e_{i}{}^{j}$ is the first interaction coefficient of i elements with j elements; [i] and [j] are the mass fraction of i elements and j elements in steel; and $a_{\left[i\right]}$ is the activity of i elements in steel. The reaction of calcium with oxygen, sulfur, and arsenic in molten steel is as follows [32,55]:

[Ca] + 2/3[As] = 1/3(Ca_3_As_2_)     ΔG^θ^ = −151,716 + 21.30 T (4)

(Ca) + 2/3[As] = 1/3(Ca_3_As_2_)     ΔG^θ^ = −41,351 − 14.29 T (5)

[Ca] + [S] = (CaS)     ΔG^θ^ = −382,559 + 112.97 T (6)

[Ca] + [O] = (CaO)     ΔG^θ^ = −491,217.93 + 146.48 T (7)

According to the standard Gibbs free energy reaction of different types of inclusions in the above steel, the following equilibrium concentration can be calculated, and the relevant derivation is as follows:

(8) $$\Delta G=\Delta G^{\theta }+RTlnK$$

(9) KCa3As2=α13Ca3As2αCa×α23As=α13Ca3As2fCa⋅wCa⋅f23As⋅w23As

(10) $$K_{CaS}=\frac{\alpha_{CaS}}{\alpha_{\left[Ca\right]}\cdot \alpha_{\left[S\right]}}=\frac{\alpha_{CaS}}{f_{\left[Ca\right]}\cdot w_{\left[Ca\right]}\cdot f_{\left[s\right]}\cdot w_{\left[s\right]}}$$

(11) $$K_{CaO}=\frac{\alpha_{CaO}}{\alpha_{\left[Ca\right]}\times \alpha_{\left[O\right]}}=\frac{\alpha_{CaO}}{f_{\left[Ca\right]}\cdot w_{\left[Ca\right]}\cdot f_{\left[O\right]}\cdot w_{\left[O\right]}}$$

Ca_3_As_2_, CaS, and CaO are considered pure substances with an activity of 1; then:

(12) $$\Delta G=\Delta G^{\theta }+RTln\frac{1}{f_{\left[Ca\right]}\cdot w_{\left[Ca\right]}\cdot f_{\left[as\right]}^{2/3}\cdot w_{\left[as\right]}^{2/3}}$$

(13) $$\Delta G=\Delta G^{\theta }+RTln\frac{1}{f_{\left[Ca\right]}\cdot w_{\left[Ca\right]}\cdot f_{\left[S\right]}\cdot w_{\left[S\right]}}$$

(14) $$\Delta G=\Delta G^{\theta }+RTln\frac{1}{f_{\left[Ca\right]}\cdot w_{\left[Ca\right]}\cdot f_{\left[O\right]}\cdot w_{\left[O\right]}}$$

where T is the temperature and the unit is K; ΔG and $\Delta G^{\theta }$ are the Gibbs free energy and the standard Gibbs free energy, respectively, in J·mol^−1^; R = 8.314 $Pa\cdot m^{3}/mol\cdot K$.

It can be seen from reaction (4) that the low temperature is beneficial for the removal of arsenic. However, an increasing temperature benefits the mass transfer of calcium and arsenic in molten iron, improving the kinetic conditions and the arsenic removal effect. The alloy is decomposed in the middle of the molten pool, and part of the released Ca vapor is transported downwards into the molten steel and undergoes a dearsenication reaction (4) in the presence of [As]. However, the dearsenication reaction between calcium and [As] in molten steel is minimal. The dearsenication reaction is mainly realized by the reaction of [Ca] dissolved in molten steel and (Ca) dissolved in slag with [As] dissolved in molten iron.

**Figure 6:**
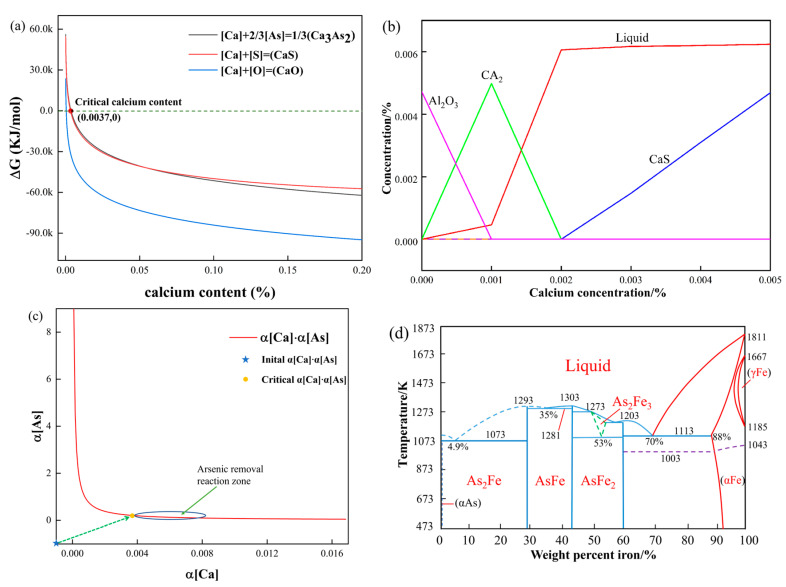
(**a**) Gibbs free energy curve of calcium content; (**b**) effect of Ca Content on inclusion precipitation in liquid steel at 1600 °C; (**c**) calcium-arsenic activity curve; (**d**) Fe-As phase diagram.

According to formulas (11)–(14), the relationship between the Gibbs free energy and calcium content of different types of calcium compounds in steel can be obtained by taking calcium content as a variable, as shown in Figure 6a. It can be seen from the figure that in this experiment, when the calcium content in molten steel is less than 0.0037%, oxygen is more likely to react with calcium, followed by sulfur, and arsenic does not react. When the calcium content increases to 0.0037%, the arsenic removal reaction can be performed, and we call this calcium content the critical calcium content for the arsenic removal reaction. Therefore, to carry out the arsenic removal reaction in molten steel, the calcium content should reach more than this value, which is the critical calcium content of the arsenic removal reaction.

Due to oxygen and sulfur in molten steel, calcium in molten steel is more likely to react with oxygen and sulfur first, so it is crucial to control the content of oxygen and sulfur to be within a narrow range. If the oxygen and sulfur content is controlled to a very low range in this experiment, the critical calcium content will also become smaller; that is, the arsenic removal reaction will be easier to carry out.

From (9) and (10), $\alpha_{\left[O\right]}$ and $\alpha_{\left[S\right]}$ can be obtained as shown in (15) and (16). The values in the formula (15) and (16) are as follows in Table 5.

(15) $$\alpha_{\left[O\right]}=f_{\left[O\right]}\cdot w_{\left[O\right]}=\frac{1}{K_{CaO}\cdot f_{\left[Ca\right]}\cdot w_{\left[Ca\right]}}$$

(16) $$\alpha_{\left[S\right]}=f_{\left[S\right]}\cdot w_{\left[S\right]}=\frac{1}{K_{CaS}\cdot f_{\left[Ca\right]}\cdot w_{\left[Ca\right]}}$$

The $\alpha_{\left[O\right]}=1.01\times 10^{-3}$, $\alpha_{\left[S\right]}=5.48\times 10^{-3}$ can be obtained by substituting the critical calcium content into formulas (15) and (16). When the calcium content in the molten steel reaches the critical calcium content in this experiment, the oxygen and sulfur concentrations in equilibrium with calcium are $w_{\left[O\right]}=0.0012\%$ and $w_{\left[S\right]}=0.0055\%$, respectively, and this activity value is the most favorable oxygen and sulfur concentration value for arsenic removal.

Arsenic mainly exists in the form of a solid solution and a compound in steel. According to the iron–arsenic phase diagram in Figure 6c [31,44], the arsenic compounds in iron mainly include As_2_Fe, AsFe, AsFe_2_, and As_2_Fe_3_. The existing form of these compounds is mainly related to the concentration of arsenic. When the arsenic content is high, arsenic will precipitate as compounds. The maximum solubility of arsenic in α-Fe is 12 wt.% at 1113 K. As the temperature decreases, the solubility of arsenic in α-Fe gradually decreases. The concentration of arsenic in molten steel is much lower than the solubility of arsenic in steel at 473 K. It can be seen that arsenic is wholly dissolved in the iron matrix.

As shown in Figure 6b, when the content of [S] is 67 ppm, the content of [Al] is 0.016%, and the temperature is 1600 °C. With the increase in the calcium content in steel, the inclusions change in the order of Al_2_O_3_ → CaO·2Al_2_O_3_ → CaS. When calcium is added at more than 0.002%, solid CaS gradually begins to precipitate.

Therefore, to achieve a good arsenic removal effect, the content of oxygen and sulfur must be controlled to a narrow range; otherwise, the added calcium is more likely to react with oxygen and sulfur, consume the arsenic removal agent, and is not conducive to arsenic removal. Therefore, this experiment used aluminum powder for the pre-deoxidation treatment. Taking into account the loss of calcium burning and gasification, the amount of arsenic removal agent added in this experiment was 10% of the steel sample.

At a temperature of 1873 K, the activity product of calcium activity arsenic is balanced, as shown in Figure 6d. This reflects the relationship between inclusions and calcium, oxygen, sulfur, and arsenic when there is a small amount of calcium in steel. Calcium is soluble in molten iron to a certain extent. The solubility of calcium in molten steel ranges from 0.030 to 0.040% at 1873 K [56]. When the dearsenication agent is added to the molten steel for dearsenication treatment, a calcium dearsenication equilibrium exists in the molten steel. According to the calculation of the arsenic removal agent added to the liquid steel process, the value of the arsenic activity in the liquid steel and the calcium arsenic activity balance is obtained. With the addition of an arsenic removal agent, the activity of calcium and arsenic in molten steel changes continuously, and the activity of arsenic in equilibrium with calcium activity decreases continuously. When the value is less than the actual arsenic activity in molten steel, inclusions of Ca_3_As_2_ begin to form.

At the same time, combined with thermodynamic calculations, it can be seen that calcium is more likely to react with oxygen, followed by sulfur, and finally, arsenic. If not deoxidized, the oxygen in the molten steel will consume a significant amount of calcium, followed by sulfur, which will also consume the calcium. Therefore, to achieve a good arsenic removal effect, it is necessary to deoxidize the molten steel and control its sulfur content.

**Figure 7:**
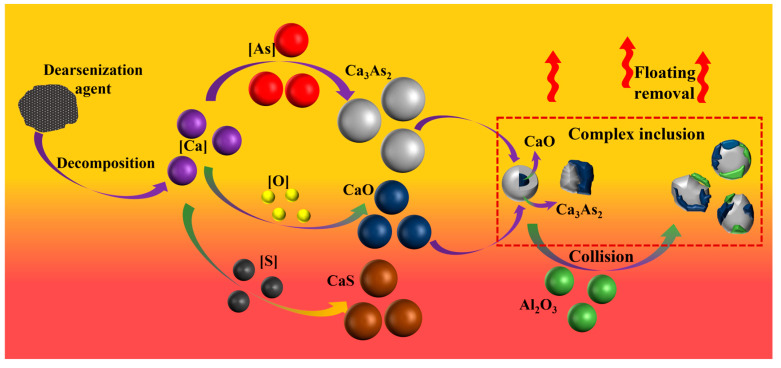
Mechanism of arsenic removal by adding arsenic removal agent into molten steel.

The primary reaction process of calcium dissolved after diffusion in molten steel is shown in Figure 7. Calcium dissolved in molten steel reacts with oxygen, sulfur, and arsenic, and its products CaO, CaS, and Ca_3_As_2_ collide and combine to form a variety of composite inclusions, followed by floating to remove them.

## 5. Conclusions

(1) The present study investigated the effectiveness of calcium-based alloys removing arsenic from molten steel. The results indicate that the optimal arsenic removal was achieved when the reaction time was 5 min. A prolonged reaction time led to an increase in arsenic content due to the decomposition of Ca_3_As_2_. Among the three calcium alloys tested, Ca-Al alloy exhibited the highest arsenic removal efficiency, with a percentage of 56.36%. The percentage of arsenic removal from calcium iron and calcium silicon alloys were 52.82% and 39.27%, respectively. These findings suggest that calcium alloys can serve as effective arsenic removal agents in steelmaking and can significantly reduce the residual arsenic content in molten steel.

(2) This study conducted thermodynamic calculations to investigate the critical calcium content necessary to initiate the dearsenication reaction in molten steel, which was found to be 0.0037%. To ensure optimal arsenic removal, it is crucial to reduce the concentrations of oxygen and sulfur in the molten steel to a narrow range. Through thermodynamic calculations, it is concluded that when the arsenic removal reaction occurs in molten steel, the concentrations of oxygen and sulfur in equilibrium with calcium are $w_{\left[O\right]}=0.0012\%$, $w_{\left[S\right]}=0.0055\%$, respectively.

(3) After dearsenication treatment with the calcium alloy, the number of inclusions in the steel samples decreased significantly. The size of the inclusions diminished, proving that calcium alloy could not only play the role of dearsenication, but could also reduce the size and number of inclusions. The dearsenication products obtained from steelmaking dearsenication experiments, utilizing calcium alloy dearsenication agents, were primarily composed of calcium aluminate, Al_2_O_3_, and other inclusions, rather than being solely formed alone. The combination of these inclusions is conducive to the floating removal and purification of molten steel. The use of calcium-based alloys can effectively minimize the arsenic content introduced by scrap steel, thereby increasing the proportion of scrap steel in converter smelting.

## Figures and Tables

**Table 1:** Typical steel standard chemical composition of special welding materials for weathering and cryogenic vessels, and the material composition selected in this experiment (wt.%).

Brand	C	Si	Mn	P	S	As
H08-3	≤0.10	≤0.08	0.30–0.55	≤0.008	≤0.003	≤0.007
Experimental base metal	0.092	0.077	0.36	0.007	0.0061	0.11

**Table 2:** Three types of calcium alloy dearsenication agent experiment changes in arsenic content change (wt.%).

	Ca-Fe Alloy		Ca-Si Alloy		Ca-Al Alloy	
Time/min	Content/%	Arsenic Removal Percentage/%	Content/%	Arsenic Removal Percentage/%	Content/%	Arsenic Removal Percentage/%
0	0.11	-	0.11	-	0.11	-
5	0.0519	52.82	0.0668	39.27	0.048	56.36
10	0.055	50.00	0.071	35.45	0.0489	55.55
15	0.0565	48.64	0.0738	32.91	0.052	52.73
20	0.0572	48.00	0.0747	32.09	0.056	49.09
25	0.057	48.18	0.076	30.91	0.0576	47.64

**Table 3:** Change in element content in molten steel (wt.%).

	No Deoxidation Experiment			Conduct Aluminum Powder Deoxidation Experiment		
	Ca-Fe	Ca-Al	Ca-Si	Ca-Fe	Ca-Al	Ca-Si
Initial O	0.0042	0.005	0.0079	0.003	0.00385	0.0053
Terminal O	0.0018	0.0018	0.0019	0.002	0.0013	0.0019
Initial S	0.0061	0.006	0.006	0.0063	0.0063	0.0063
Terminal S	0.0004	0.0004	0.0004	0.0004	0.0004	0.0004
Initial As	0.11	0.11	0.11	0.11	0.11	0.11
Terminal As	0.082	0.081	0.096	0.0519	0.048	0.0668
Arsenic removal percentage	25.45	26.36	12.73	52.82	56.36	39.27

**Table 4:** Solute interaction coefficient at 1873 K.

i	j							
	C	Mn	O	S	P	Si	As	Ca
O	−0.45	−0.021	−0.2	−0.127	0.07	−0.131	-	-
S	0.11	−0.012	−0.27	−0.028	-	0.08	0.0041	−0.931
Ca	−0.34	−0.1	-	0	-	−0.097	-	−0.002
As	0.25	−0.029	-	0	0.1124	0.224	0.11	-

**Table 5:** Thermodynamic calculation values.

$f_{\left[Ca\right]}$	$f_{\left[O\right]}$	$f_{\left[S\right]}$	$w_{\left[Ca\right]}$	$K_{CaO}$	$K_{CaS}$
0.8421	0.8712	1.0045	0.0037	316,225	58,530

## Data Availability

No data were used for the research described in the article.

## References

[1] Liang T., Wang S., Lu C., Jiang N., Long W., Zhang M. (2020). Environmental impact evaluation of an iron and steel plant in China: Normalized data and direct/indirect contribution. J. Clean. Prod..

[2] Andonovski G., Tomažič S. (2022). Comparison of data-based models for prediction and optimization of energy consumption in electric arc furnace (EAF). IFAC-PapersOnLine.

[3] Zhang X., He Y., Li M., Hu X. (2022). The study of heat-mass transfer characteristics and multi-objective optimization on electric arc furnace. Appl. Energy.

[4] Marulanda-Durango J., Escobar-Mejía A., Alzate-Gómez A., Álvarez-López M. (2021). A Support Vector machine-Based method for parameter estimation of an electric arc furnace model. Electr. Power Syst. Res..

[5] Kumar B., Roy G.G., Sen P.K. (2020). Comparative exergy analysis between rotary hearth furnace-electric arc furnace and blast furnace-basic oxygen furnace steelmaking routes. Energy Clim. Chang..

[6] Brandner U., Antrekowitsch J., Leuchtenmueller M. (2021). A review on the fundamentals of hydrogen-based reduction and recycling concepts for electric arc furnace dust extended by a novel conceptualization. Int. J. Hydrogen Energy.

[7] Kim J., Son M., Park J., Kim J. (2022). Optimized rotary hearth furnace utilization with blast furnace and electric arc furnace: Techno-economics, CO_2_ reduction. Fuel Process Technol..

[8] Tian B., Wei G., Li X., Zhu R., Bai H., Tian W. (2022). Effect of hot metal charging on economic and environmental indices of electric arc furnace steelmaking in China. J. Clean. Prod..

[9] Cheng R.J., Ni H.W., Zhang H., Zhang X.K., Bai S.C. (2017). Mechanism research on arsenic removal from arsenopyrite ore during a sintering process. Int. J. Miner. Metall. Mater..

[10] Su Y., Huang W., Liu Y., Chang C., Kuo Y. (2017). Utilization of electric arc furnace dust as regenerable sorbents for the removal of hydrogen sulfide. Ceram. Int..

[11] Kicińska A. (2019). Environmental risk related to presence and mobility of As, Cd and Tl in soils in the vicinity of a metallurgical plant—Long-term observations. Chemosphere.

[12] Potysz A. (2023). Microbial influence and dynamics of metallurgical waste dissolution in a landfill and recovery context: A multi-phase experimental approach and geochemical model. Chemosphere.

[13] Liu M., Ma G., Zhang X., Liu J., Wang Q. (2020). Preparation of Black Ceramic Tiles Using Waste Copper Slag and Stainless Steel Slag of Electric Arc Furnace. Materials.

[14] Maecki S., Gargul K., Warzecha M., Stradomski G., Hutny A., Madej M., Dobrzyński M., Prajsnar R., Krawiec G. (2021). High-Performance Method of Recovery of Metals from EAF Dust—Processing without Solid Waste. Materials.

[15] Moskal M., Migas P., Karbowniczek M. (2022). Multi-Parameter Characteristics of Electric Arc Furnace Melting. Materials.

[16] Cao R., Ma H., Yi L., Zhang J., Cui R.J. (1998). Effect of Arsenic, Tin and Antimony on Hot Plasticity of Steel 35Mn. Spec. Steel.

[17] Liu S., Sun S. (2001). A study on dearsenication of molten iron and liquid steel with Ca-Si alloy. Spec. Steel.

[18] Xu X. (2005). The physico-chemical characteristics of arsenic in metallurgical process and their influence on the steel during the hot rolling. Shonghai Steel Iron Res..

[19] Wang H., Kang J., Wang Y. (2022). Discovery and identification of arsenic removal products from molten steel by adding rare earth. J. Mater. Res. Technol..

[20] Wang H., Bai B., Jiang S., Sun L., Wang Y. (2019). An in situ Study of the Formation of Rare Earth Inclusions in Arsenic High Carbon Steels. ISIJ Int..

[21] Zhu Y.Z., Xu J.P. (2012). A method to study interface diffusion of arsenic into a Nb-Ti microalloyed low carbon steel. Int. J. Miner. Metall. Mater..

[22] Cheng R., Zhang H., Ni H. (2019). Arsenic Removal from Arsenopyrite-Bearing Iron Ore and Arsenic Recovery from Dust Ash by Roasting Method. Processes.

[23] Paz-Gómez D.C., Pérez-Moreno S.M., Gázquez M.J., Guerrero J.L., Ruiz-Oria I., Ríos G. (2021). Arsenic removal procedure for the electrolyte from a hydro-pyrometallurgical complex. Chemosphere.

[24] Li Y., Qi X., Li G., Duan X., Yang N. (2022). Removal of arsenic in acidic wastewater using Lead-Zinc smelting slag: From waste solid to As-stabilized mineral. Chemosphere.

[25] Marcińczyk M., Ok Y.S., Oleszczuk P. (2022). From waste to fertilizer: Nutrient recovery from wastewater by pristine and engineered biochars. Chemosphere.

[26] Khanam T., Liang S., Xu S., Musstjab Akber Shah Eqani S.A., Shafqat M.N., Rasheed H., Bibi N., Shen H., Zhang J. (2023). Arsenic exposure induces urinary metabolome disruption in Pakistani male population. Chemosphere.

[27] Lin L., Zeng J.Q. (2021). Consideration of green intelligent steel processes and narrow window stability control technology on steel quality. Int. J. Miner. Metall. Mater..

[28] Tshilombo K. (2010). Determination of inclusions in liquid steel after calcium treatment. Int. J. Min. Met. Mater..

[29] Li C.X. (2019). Effect of La on the Precipitation of Inclusions in As-Bearing High Carbon Steel. Master’s Thesis.

[30] Xiong L. (2019). Study of Effect of Arsenic and Lanthanum on Properties of High Carbon Steel. Master’s Thesis.

[31] Xin W. (2015). Effect of Arsenic on the Properties of Steel and Improvement by Adding Rare Earth. Ph.D. Thesis.

[32] Li W. (2016). The Applied Fundamental Research on the Removal of Residual Element Arsenic during Steelmaking Process. Ph.D. Thesis.

[33] Zhang T., Li Z., Young F., Kim H.J., Tillmann W. (2014). Global Progress on Welding Consumables for HSLA Steel. ISIJ Int..

[34] Lan F., Zhuang C., Li C., Yang G., Yao H. (2021). Effect of Calcium Treatment on Inclusions in H08A Welding Rod Steel. Met. Open Access Metall. J..

[35] Nakamura Y., Tokumitsu N., Harashima K., Segawa K. (1976). Refining of 18%Cr–8%Ni Steel with Ca-CaF_2_ Solution. Trans. Iron Steel Inst. Jpn..

[36] Katayama H., Kajioka H., Harashima K.U., Inatomi M. (1979). Dephosphorization of High Chromium Molten Steel with CaC_2_-CaF_2_ Flux. Trans. Iron Steel Inst. Jpn..

[37] Zhu Y.K. (1986). Dearsenification of Hot Metal with CaC_2_-CaF_2_ Slag. J. Beijing Iron Steel Technol..

[38] Zhu Y.K., Dong Y.C., Peng Y.Q., Wei S.K. (1985). A Study of the Chemical Equilibrium between Arsenic and Calcium in Steel Melt. Iron Steel.

[39] Dong Y., Shi Z., Zhang L., Peng Y., Hong Y. (1984). Study on dearsenization of molten iron. Gangtie.

[40] Li W.B., Bao Y.P., Wang M., Lin L. (2015). Analysis of factors for Si-Ca-Ba alloy+CaF_2_ dearsenication of molten steel. Iron Steel.

[41] Wang H., Yu P., Jiang S., Wang Y. (2020). Effect of Heterogeneous Nucleation on Removal of Arsenic from Molten Steel by Rare Earth Addition. Met. Open Access Metall. J..

[42] Lan F., Zhuang C., Li C., Yang H., Yang G., Yao H., Zhang Z. (2023). Effect of Rare-Earth Cerium on Nonmetallic Inclusions in Fe–Mn–C–Al Twinning-Induced Plasticity Steel. Steel Res. Int..

[43] Wang J., Luo L., Kong H., Zhou L. (2011). The Arsenic Removal from Molten Steel. High Temp. Mater. Process..

[44] Liang Y. (1983). The influence of arsenic (as) on the physical properties of carbon steel. Gangtie.

[45] Xian A. (1999). Impurities in steel and their influence on steel properties. Iron Steel.

[46] Lan Y., Sridhar S. (2011). Effects of Residual Elements Arsenic, Antimony, and Tin on Surface Hot Shortness. Metall. Mater. Trans. B.

[47] Wang X., Tang X.P., Yang J.M., Lei Y. (2011). Characteristics and Effect of Impurity Elements in Steel. Spec. Steel Technol..

[48] Wang H., Xiong L., Zhang L., Wang Y., Shu Y., Zhou Y. (2017). Investigation of RE-O-S-As Inclusions in High Carbon Steels. Metall. Mater. Trans. B..

[49] Verma N., Pistorius P.C., Fruehan R.J., Potter M., Story S.R. (2011). Transient Inclusion Evolution During Modification of Alumina Inclusions by Calcium in Liquid Steel: Part II. Results and Discussion. Metall. Mater. Trans. B..

[50] Wang H., Jiang S., Yu P., Bai B., Wang Y. (2020). Distribution of Arsenic Inclusions in Rare Earth Steel Ingots. Met. Open Access Metall. J..

[51] Xin W., Song B., Song M., Song G. (2015). Effect of Cerium on Characteristic of Inclusions and Grain Boundary Segregation of Arsenic in Iron Melts. Steel Res. Int..

[52] Dong Y., Wei S.K., Peng Y.Q., Zhu Y.K. (1986). Activity of as in Fe-As-C-j Melts. Acta Metall. Sin..

[53] Zhuang C., Liu J., Mi Z., Jiang H., Tang D., Wang G. (2014). Non-Metallic Inclusions in TWIP Steel. Steel Res. Int..

[54] Shi C.B., Chen X.C., Guo H.J. (2012). Characteristics of inclusions in high-Al steel during electroslag remelting process. Int. J. Miner. Metall. Mater..

[55] Liu S.P. (2000). Study on Thermodynamic Properties of Iron-Arsenic Melt and Arsenic Removal from Molten Iron and Molten Steel. Ph.D. Thesis.

[56] Janke D., Zhongting M.A., Valentin P. (2000). Improvement of Castability and Quality of Continuously Cast Steel. ISIJ Int..

